# Systematic Approach to Reducing Errors in Deoxynivalenol Quantification: Insights from Bulk Wheat Sampling and Sample Preparation

**DOI:** 10.3390/toxins18010013

**Published:** 2025-12-24

**Authors:** Li Li, Bingjie Li, Jin Ye, Di Cai, Yu Wu, Peng Li, Bing Zhang, Jie Wang, Xiujuan Li, Yi Shao, Songxue Wang

**Affiliations:** 1 Academy of National Food and Strategic Reserves Administration, NFSRA Key Laboratory of Grain and Oil Quality and Safety, Beijing 100037, China; ll@ags.ac.cn (L.L.); haiyangzhixin2006@163.com (B.L.); yj@ags.ac.cn (J.Y.); cd@ags.ac.cn (D.C.); wyu@ags.ac.cn (Y.W.); lp@ags.ac.cn (P.L.); zb@ags.ac.cn (B.Z.); 2 College of Food Science and Technology, Nanjing Agricultural University, 1 Weigang Road, Nanjing 210095, China; 2023808109@stu.njau.edu.cn; 3 Grain and Oil Product Quality Supervision and Inspection Station of Xinjiang Uygur Autonomous Region, Urumqi 830049, China; lixj@ags.ac.cn; 4 NHC Key Laboratory of Food Safety Risk Assessment, China National Center for Food Safety Risk Assessment, Beijing 100021, China; 5 School of Public Health, Southern Medical University, Guangzhou 510515, China

**Keywords:** wheat, deoxynivalenol, sampling, sample preparation, potential errors

## Abstract

Accurate quantification of Deoxynovalienol (DON) in wheat is critical for food safety, but current methods suffer from poor reproducibility due to inconsistent operational parameters across the sampling and analysis workflow. To address this issue, this study focused on truck-loaded bulk wheat and conducted a comprehensive analysis covering the entire process from sampling to laboratory testing. By examining parameters at each stage—test portion, laboratory sample, composite sample, and primary sample—and applying the Monte Carlo simple random sampling principle, the variability associated with the full-process parameters for DON detection in wheat was systematically analyzed. The errors introduced at each step were evaluated, leading to the development of a representative measurement procedure for DON in truck-loaded bulk wheat. The results indicate that for truck-loaded bulk wheat, sampling should be conducted using a random distribution method with no fewer than 11 sampling points, each providing a primary sample of at least 500 g. The composite sample should be homogenized three times using a cone-and-quartering divider before subsampling. The laboratory sample should weigh no less than 750 g and be ground to a particle size of 1 mm. After thorough mixing of the ground sample, 5 g should be accurately weighed for analysis. This measurement procedure introduces a total relative error of 12.9%. The proposed protocol for DON detection in truck-loaded wheat offers a practical approach that minimizes error contribution from each parameter while maintaining low economic and time costs, ensuring feasibility for field implementation.

## 1. Introduction

Deoxynivalenol (DON), also known as vomitoxin, is a trichothecene mycotoxin primarily produced by Fusarium graminearum and Fusarium culmorum. DON exhibits strong cytotoxic, immunotoxic, and teratogenic properties, posing a significant threat to human and animal health [1,2]. It is widely detected in grains such as wheat, corn, and barley as well as in their processed derivatives, including flour, cereal, and feed [3,4]. Wheat is a major source of plant-derived calories and proteins in the human diet, and is particularly susceptible to DON contamination [5,6]. Field-related biotic and abiotic stresses (e.g., disease, heat stress) can alter wheat physiology, increasing susceptibility to Fusarium infection and thereby elevating the risk of DON contamination [7]. Reports indicate that 60–70% of the global wheat supply is contaminated with DON, leading to substantial economic losses. In response, many countries have established maximum limits for DON in wheat and wheat-derived products [8,9,10]. Therefore, precise quantification of DON levels in wheat batches is essential for food safety and regulatory compliance.

**Figure 1:**
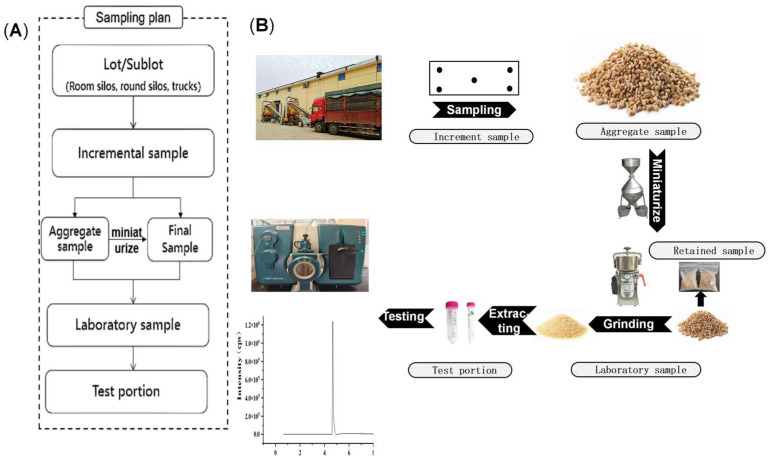
Measurement procedure for DON in bulk wheat. Panel (**A**) outlines each procedural step and Panel (**B**) describes the workflow for DON analysis in on-board bulk wheat, from sampling to sample preparation and testing.

The procedure for DON quantification in wheat grains consists of three critical steps, sampling, sample preparation, and detection (Figure 1), with detection serving as the final analytical step. DON detection technologies include colloidal gold test strips [11], enzyme-linked immunosorbent assays [12], fluorescence immunoassays [13], liquid chromatography, and liquid chromatography–mass spectrometry [14,15,16,17]. Advances in analytical technology and deeper research have progressively enhanced the accuracy and precision of DON detection. However, due to the inherently heterogeneous distribution of mycotoxins in grains, the influence of sampling and sample preparation on measurement accuracy is substantial. Whitaker [18,19,20] demonstrated that measurement errors predominantly originate from the sampling process (88%), followed by sample preparation (10%), while laboratory analysis contributes only 2%, underscoring the importance of sampling and preparation in the overall measurement procedure. Wheat may become contaminated with DON at various stages: during field growth, harvesting, or storage [21]. Such contamination may occur in individual grains or may be localized within specific areas of a batch, resulting in a highly heterogeneous DON distribution and complicating accurate quantification. Consequently, establishing scientifically sound, cost-effective, and representative sampling procedures is essential. The primary international sampling standards for mycotoxins include Codex Alimentarius CXS 193 [22], International Organization for Standardization ISO 24333 [23], and European Union Regulation (EC) No 401/2006 [24]. However, notable discrepancies exist between these standards. For example, CXS 193 provides detailed methodologies for sampling and sample preparation, along with guidelines for interpreting the test results. ISO 24333 specifies sampling procedures and associated equipment but lacks explicit requirements for sample preparation and detection. (EC) No. 401/2006 outlines the sampling parameters and performance criteria for detection methods but does not provide detailed specifications for sample preparation. Additionally, while both CXS 193 and ISO 24333 specify a laboratory sample size of 1 kg for DON analysis in wheat, they differ in sample collection methods: CXS 193 recommends collecting 100 g per sampling point from 10 points, whereas ISO 24333 requires reducing an aggregate sample to yield a 1 kg laboratory sample. In contrast, (EC) No 401/2006 recommends a 10 kg laboratory sample. These discrepancies highlight the inconsistencies and gaps in existing sampling standards, complicating their selection and practical implementation.

Current research on the sampling of DON in wheat remains limited. Hart and Schabenberger [25] analyzed the variability of DON levels in truckloads of wheat during a wheat scab epidemic year, focusing primarily on variability within the sampling and analysis process. Whitaker et al. investigated the variability associated with sampling, sample preparation, and analysis in DON detection [26]. Their study measured the variability of DON testing in wheat using a 0.454 kg sample, a Romer mill, a 25 g comminuted subsample, and the Romer Fluoroquant analytical method. Unlike other mycotoxin testing procedures, this study indicated that sampling variation was not the largest source of error. However, the parameters investigated at each stage were limited in scope. A study on the distribution of DON and ochratoxin A (OTA) contamination within a 26 t truckload of wheat kernels suggested that at high contamination levels (>2.0 ppm), the number of sampling points for DON could be reduced to 5–10 [27]. The study also highlighted that sampling errors were less significant than those introduced during sample preparation. However, sample preparation parameters were examined under a single condition (particle size of 0.5 mm) without considering the practical constraints of on-site screening and laboratory sample preparation conditions. In China, large-scale wheat intake during the harvest season presents additional challenges. Grain storage enterprises may receive dozens or even hundreds of wheat truckloads daily, each exceeding 20 t. Consequently, technicians must complete the entire process, from sampling to detection, within a limited timeframe. To improve efficiency, some storage enterprises reduce the laboratory sample size to less than 1 kg. However, systematic research on critical parameters such as particle mixing methods, minimum laboratory sample size, grinding particle size, and test portion size remains lacking. These factors significantly impact detection accuracy and, consequently, influence decisions regarding the acceptance or rejection of batch or sub-batch samples. Despite the recognized importance of sampling and sample preparation, existing studies have largely focused on optimizing isolated steps or individual parameters. There remains a gap in holistic and systematic research that traces, quantifies, and integrates errors throughout the entire workflow—truck sampling to the preparation of laboratory test portions. Particularly in the context of China’s typical bulk grain storage and transportation scenarios, the development of a standardized, operationally feasible, and error-controlled protocol continues to be a critical unresolved issue.

This study focuses on DON measurement in wheat truckloads, which represent the largest testing volume, and comprehensively investigates the entire process from sampling to laboratory detection. A representative measurement procedure for DON quantification in wheat truckloads is proposed by analyzing the parameters at each stage and evaluating the errors introduced using relative standard deviation estimates. This study clearly defines the parameters and their associated errors at each stage of the measurement procedure, selecting optimal parameter settings based on acceptable error ranges. This provides the industry with an accurate, reproducible, and practical standardized operational protocol.

## 2. Results and Discussion

### 2.1. Number of Sampling Points and Primary Sample Size Evaluation

**Figure 2:**
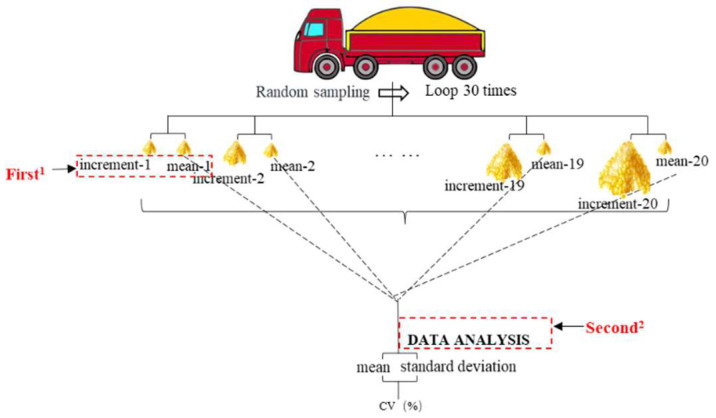
Flowchart of the sampling-point formation process. 1: The first sampling process—1 to 30 points were randomly selected from the 200 wheat DON values across 30 rounds of sampling. The mean values from each round formed a dataset corresponding to the number of sampling points. 2: The second sampling process—From this dataset, two values were randomly selected with replacement 30 times, and the coefficient of variation (CV) for the mean was calculated. A boxplot of the CV values for 1–20 sampling points was then created.

Descriptive statistical analysis of the raw data used to evaluate the number of sampling points is presented in Table S1. When the number of sampling points (sample size) was 200, and the primary sample size was 100 g, the mean DON concentration was 675.2 µg/kg, with a relative standard deviation (RSD) of 66.3%. The large difference between the mean and median indicates considerable influence from outliers. Increasing the primary sample size to 300 and 500 g raised the mean values to 740.1 and 753.8 µg/kg, respectively, for the 200 sampling points, while decreasing RSD to 42.5% and 34.0%, respectively. The reduced discrepancy between the mean and median, along with the smaller data fluctuation range, suggests improved measurement stability. More than 50% of the measurements fell below the average of the 200 measurements, suggesting that the DON detection results followed an approximately normal distribution. The concentration data were tested for normality using the Shapiro–Wilk test (W = 0.36, *p* = 0.11), indicating that the data approximately conform to a normal distribution. This trend aligns with the findings of Whitaker et al. regarding the variability in sampling, sample preparation, and DON analysis in wheat [26]. To further examine the effect of sampling points, we employed a Monte Carlo resampling estimation approach (the same fundamental principle was applied to all subsequent parameter studies). From the 200 wheat DON detection data points, 1–20 data points were randomly selected for 30 rounds of sampling. The mean values from each round formed a dataset representing different sampling point counts. From this dataset, two values were randomly selected with replacement 30 times, and the coefficient of variation (CV) of the mean was calculated. A boxplot of the CV values for 1–20 sampling points was generated (Figure 2).

**Figure 3:**
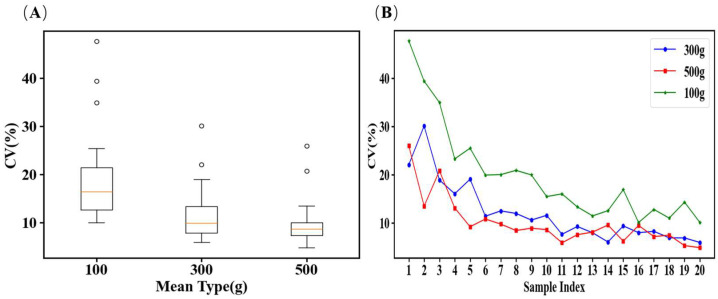
Boxplot and mean CV values for 30 datasets at different primary sample sizes. (**A**): boxplot of CV values for 1–20 sampling points. B: line graph of the average RSD for 30 datasets at different sampling points with primary sample sizes of 100, 300, and 500 g. (Boxplot explanation: The lower boundary of the box represents the first quartile (Q1), or the 25th percentile; the line inside the box represents the median (Q2), or the 50th percentile; the upper boundary of the box represents the third quartile (Q3), or the 75th percentile; the length of the box itself represents the interquartile range (IQR), which is calculated as Q3 − Q1 and contains the middle 50% of the data; the whiskers show the range of the majority of the dataset: the upper whisker extends to the maximum data point that lies within Q3 + 1.5 × IQR, while the lower whisker extends to the minimum data point that lies within Q1 − 1.5 × IQR; Outliers: any individual datapoints that fall outside the range of the whiskers (i.e., beyond Q3 + 1.5 × IQR or below Q1 − 1.5 × IQR) are plotted individually as dots (or sometimes asterisks) and are considered outliers. (**B**): the line graph of the average CV values for 30 datasets across different sampling point counts with primary sample sizes of 100, 300, and 500 g. The entire data processing workflow was implemented using a compact computational program developed in Python (version 3).

The resulting boxplot of the CV values for 200 sampling points (Figure 3A) showed that as the primary sample size increased from 100 to 500 g, the variability range narrowed, indicating that larger primary sample sizes effectively reduce sampling error for a fixed number of sampling points. Figure 3B presents the line graph of the average CV values for 30 datasets across different sampling point counts with primary sample sizes of 100, 300, and 500 g. For a given primary sample size, the RSD of the measurement results gradually decreased and eventually stabilized as the number of sampling points increased. This confirms that increasing the number of sampling points in bulk wheat sampling significantly enhances the accuracy and reliability of grain quality testing. Cheng and Stasiewicz [28] reported that increasing the number of sampling points improved the accuracy of aflatoxin estimation in corn, a finding consistent with studies on other mycotoxins, such as ochratoxin A [29,30,31]. However, our results indicate that DON contamination in wheat is more uniformly distributed than other mycotoxins, allowing the number of sampling points to be reduced without compromising accuracy. This observed spatial structure differs from the random distribution reported by Rivas Casado et al. [32], providing new evidence on the spatial heterogeneity of DON in bulk wheat. When the number of sampling points exceeded 11, the contribution of additional points in reducing the sampling error became negligible. When the primary sample size was 100 g, the CV values across the different sampling point numbers were significantly larger than those for 300 and 500 g. Moreover, for primary sample sizes of 300 and 500 g, the CV values exhibited minimal variation across different sampling point counts. Therefore, it is recommended that the primary sample size should not be less than 300 g.

### 2.2. Number of Mixing Cycles Evaluation

During fieldwork, harvesting, and storage, grains are susceptible to fungal infections, which may be concentrated in specific grains or localized areas within the grain heap. This results in a heterogeneous distribution of mycotoxins across the batch [32,33,34]. Because of this uneven distribution, complete homogenization of the target analytes through grain mixing alone is impossible. However, mixing remains an essential step, as it promotes a more uniform distribution of grain particles and ensures the randomness and representativeness of subsequent sample preparation. Previous studies have shown that many sampling plans involve direct subsampling after sampling, which can lead to samples that do not accurately represent the DON content of the entire wheat batch. Table S2 presents the mixing results for dyed wheat at different positions.

The uneven distribution of mycotoxins in wheat grains can be attributed to two primary factors: First, grain size, density, and weight differences contribute to particle movement variations. Research indicates that optimizing sampling protocols based on the physical characteristics of grains (particle size and density) can effectively enhance the homogeneity of organic wheat lots, thereby reducing sampling variability in the quantitative analysis of deoxynivalenol contamination [35]. Second, these physical differences cause lighter grains near the edges to move differently when passing through a divider, leading to stratification within the wheat mass. The results indicate that as the number of mixing cycles increased, the distribution of dyed wheat within the sample became more uniform. After the first mixing cycle, the upper section exhibited the poorest uniformity, followed by the middle and bottom sections. This may be attributed to the reduced mobility of the contaminated grains in the upper section, resulting in a limited mixing efficiency. Conversely, the grains at the bottom, influenced by gravity, tended to move slower as they passed through the divider, which enhanced mixing efficiency. However, after more than three mixing cycles, the RSD values among the wheat grains stabilized, and further mixing yielded negligible improvements in uniformity. Therefore, at least three mixing cycles are recommended to achieve acceptable uniformity.

### 2.3. Laboratory Sample Evaluation

Given the varying requirements for laboratory sample sizes across different sampling and detection standards, in this study, we assessed the minimum laboratory sample size required for accurate DON detection in wheat grains.

**Figure 4:**
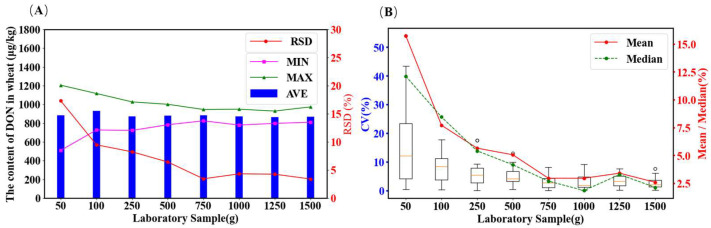
Analysis of laboratory sample volume inspection results. (**A**) the mean, maximum, minimum, and relative standard deviation (RSD) values of the DON assay results obtained from different laboratory sample sizes. (**B**) boxplot of the CV values for different laboratory sample sizes. The entire data processing workflow was implemented using a compact computational program developed in Python (version 3).

As shown in Figure 4A, after mixing the wheat grain samples, the mean DON concentration across different laboratory sample sizes was 900 µg/kg. As the laboratory sample size increased, the difference between the maximum and minimum values within the samples gradually decreased. When the laboratory sample size exceeded 750 g, the RSD values and the difference between extreme values stabilized, with the deviation in extreme values decreasing from 91.9% to less than 21.2%. These results indicate that increasing the laboratory sample size improves measurement consistency and reduces dispersion [33]. To further evaluate the laboratory sample size variability, two values were randomly selected from the dataset for 30 rounds of laboratory sample size assessment, and the CV of the mean was calculated. A boxplot of CV values for different laboratory sample sizes was generated (Figure 4B). Statistical analysis was performed on the mean, median, and interquartile range (Q3–Q1) of the boxplots. The upper and lower boundaries were calculated using Q1 − 1.5 × IQR and Q3 + 1.5 × IQR.

As shown in Figure 4B and Table 1, increasing the laboratory sample size from 50 to 1500 g resulted in a significant decrease in the mean and median CV values. For example, the average CV value for a 50 g sample was 15.75%, whereas the average CV value for a 1500 g sample was reduced to only 2.61%, representing a relative reduction of 83.4%. This indicates that larger sample sizes substantially minimize measurement error. When higher measurement uncertainty is acceptable (e.g., CV > 10%), a sample size of 100 g may suffice. However, when a lower measurement error is required (e.g., CV < 5%), a laboratory sample size of no less than 750 g is recommended. Ultimately, selecting an appropriate laboratory sample size requires balancing accuracy requirements with practical resource constraints. For measurements requiring higher precision, a sample size of 750 g or greater is advisable.

### 2.4. Grinding Particle Size Evaluation

**Figure 5:**
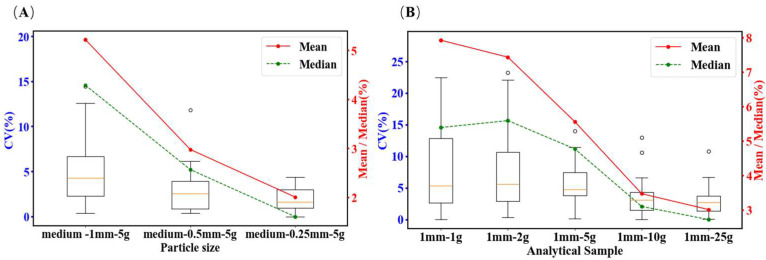
Boxplot of CV values under different crushing particle sizes and weighing volumes. (**A**) Boxplot of the coefficient of variation (CV) values of wheat DON measurements under different crushing sizes (fixed sample weight). (**B**) Boxplot of the CV values of wheat DON measurements under different sample weights (fixed grinding size). The entire data processing workflow was implemented using a compact computational program developed in Python (version 3).

Because fungal infection and mycotoxin contamination predominantly occur in the outer layers of wheat kernels [36,37], removing the most contaminated outer layer of the grain, which accounts for 7–10% of the total mass, can eliminate 50–60% of DON contamination. Cheli et al. [38] further indicated that grinding factors significantly influence the DON content, as the distribution of DON within milling fractions is not uniform. Therefore, the sample preparation process in the laboratory is critical because the grinding particle size plays a significant role in the accuracy of the test results. The CXS 193 specifies a grinding particle size of 0.85 mm, whereas ISO 24333 and (EC) No. 401 require the homogenization of laboratory samples without specifying the exact parameters. In contrast, GB 5009.111 [10] defines a grinding particle size of 0.5–1.0 mm. These inconsistencies highlight the need for systematic evaluation. To assess the impact of grinding particle size, two values were randomly selected from the dataset for 30 rounds of analysis, and the CV for the mean was calculated. A boxplot of the CV values for the different laboratory sample sizes (1.0, 0.5, and 0.25 mm) is presented in Figure 5A. In this study, the CV values were analyzed for different grinding particle sizes. As shown in Table S3, as the particle size decreased from 1.0 to 0.25 mm, the mean and median CV values gradually declined. For example, the average CV for a 1.0 mm particle size was 5.22%, whereas for a 0.25 mm particle size, it was reduced to 2.01%. This indicates that smaller particle sizes help minimize measurement variability. Therefore, finer particles should be used when high precision is required.

### 2.5. Sample Weighing Amount Evaluation

Because of the uneven distribution of DON within milling fractions, larger sample weights generally improve the representativeness of the measurement results, thereby reducing the risks associated with the heterogeneity of the powdered samples. However, practical constraints—such as onsite screening conditions, extraction equipment limitations, processing time, and solvent consumption—necessitate selecting an appropriate sample weight. The CXS 193 standard recommends a sample weight of 25 g for DON detection in grains. In contrast, ISO 24333-2009 and (EC) No 401/2006 do not specify a recommended sample mass. The GB 5009.111 standard prescribes sample weights of 2 g for mass spectrometry and 25 g for liquid chromatography.

Rapid screening methods commonly employed in China—including enzyme-linked immunosorbent assays (ELISA), colloidal gold assays, and time-resolved fluorescence assays—typically employ sample weights of 5–10 g or lower to facilitate rapid high-throughput screening and meet the timeliness requirements of grain storage and transportation sites. Therefore, the impact of different sample weights on the detection results was investigated using a grinding particle size of 1.0 mm. Two values from each dataset were randomly selected for 30 sampling rounds of sample weight analysis and the CV of the mean was calculated. A boxplot of the CV values for different sample weights was generated (Figure 5B) and the results were statistically analyzed. The mean and median CV values gradually decreased with increasing sample weight, indicating that a larger sample weight reduces measurement variability. For example, when the sample weight was 1 g, the mean and median CV values were relatively high, with a significant difference between the two, and longer box lengths. The error range for 50% of the data was between 2.67% and 12.86%, while the 95% error range extended from 0% to 28.15%, reflecting considerable data variability. In contrast, at a sample weight of 25 g, both the mean and median CV values were lower and closer to each other, with a shorter box length, indicating improved data stability.

These findings provide practical guidance for selecting an appropriate sample weight within an acceptable error range, while balancing measurement accuracy and processing efficiency. Under the conditions of a 1.0 mm grinding particle size and thorough sample mixing, a sample weight of at least 5 g is recommended.

### 2.6. Error Analysis

**Figure 6:**
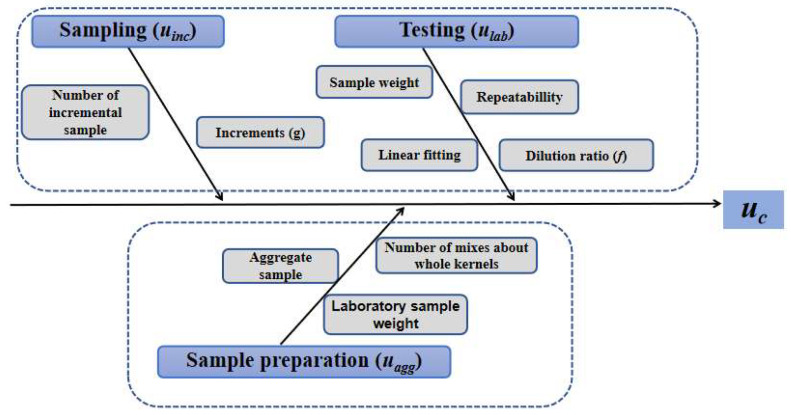
Fishbone diagram of error sources for DON determination. The error sources include those introduced during the sampling, sample preparation, and detection stages.

Throughout the measurement procedure—including sampling, sample preparation, and detection—errors are unavoidable. Therefore, the precision (error) of the entire workflow was evaluated by analyzing the experimental results for the parameters optimized at each stage. The primary error sources for DON determination in wheat arise from sampling, sample preparation, and testing, as presented in Figure 6.

The average DON concentration in a batch of wheat samples is calculated as follows:

(1) $$r=\rho \times f_{inc}\times f_{agg}\times f_{lab}$$

where

*r* is the average DON content in the wheat sample batch (mg/kg);

*ρ* is the mass concentration of DON (mg/kg);

*f_inc_* is the sampling correction factor (initial value = 1);

*f_agg_* is the sample preparation correction factor (initial value = 1);

*f_lab_* is the detection correction factor (initial value = 1).

The combined uncertainty introduced by the entire process is calculated as follows:

(2) $$u=\sqrt{u_{inc}^{2}+u_{agg}^{2}+u_{lab}^{2}}$$

where

*u* is the relative combined standard uncertainty for DON determination in a batch of wheat samples;

*u_inc_* is the relative uncertainty associated with sampling;

*u_agg_* is the relative uncertainty associated with sample preparation;

*u_lab_* is the laboratory measurement uncertainty (not separately added to avoid double-counting, as it is inherently included in all measure results).

The sampling, sample preparation, and testing stages may systematically affect the accuracy and repeatability of DON content assessment in wheat batches. The primary mechanism of this influence is the cumulative propagation of measurement errors. To address this issue, we introduced correction factors for three key process parameters (all with initial values of 1) in Equation (1). By parameterizing the error contributions of each stage, the combined uncertainty was computed via Equation (2).

Error synthesis followed the standard uncertainty approach, which includes three calculation steps: first, the error components of each independent stage were squared, then algebraically summed, and finally, the combined error estimate value was obtained through a square-root operation. Notably, the experimental data showed skewed distributions; however, based on the mathematical principles of the Central Limit Theorem, a normal distribution assumption is still used in error analysis during statistical inference. To ensure the systematic reliability of the statistical quantities, the mean CV of the detection results of each process stage was selected as the benchmark error index for the systematic error analysis. Considering that each stage of the process involves detection and that all results inherently reflect the detection error, the formula does not separately account for the detection error. This analysis primarily focuses on errors associated with the sampling and sample preparation stages.

For example, when evaluating the DON content in a wheat batch near the regulatory limit (~1000 µg/kg), using 11 sampling points with a primary sample size of 500 g, three mixing cycles, a 750 g laboratory sample size, a grinding particle size of 1.0 mm, and a sample weight of 5 g, the total error introduced by the entire measurement procedure is 12.9%, with an expanded uncertainty of 25.8% (95% confidence interval, k = 2).

According to our findings, if the sampling requirements of the CXS 193 standard are followed—10 sampling points, a primary sample size of 100 g, a laboratory sample size of 1000 g, a grinding particle size of 0.85 mm, and a sample weight of 25 g—the total error introduced by the entire measurement procedure is 20.3%, with an expanded uncertainty of 40.6% (95% confidence interval, k = 2). The difference in error between the CXS 193 standard and our approach primarily arises from the primary sample size (Table 1); using 500 g instead of 100 g (CXS 193) reduces the sampling error from 19.9% to 8.6%. Therefore, under a similar number of sampling points, the primary sample size at each point is the most influential factor affecting the overall measurement error. The sampling scheme proposed in this study demonstrates superior performance in terms of accuracy, speed, and cost effectiveness for DON detection in bulk wheat during transit. When applying this sampling approach to long-term or specialized storage scenarios (e.g., controlled-environment warehouses or pest-prone facilities), adjustments must incorporate actual storage conditions, monitoring data, and temporal-environmental covariates in the design phase.

## 3. Conclusions

In this study, we systematically evaluated the parameters influencing the sampling, sample preparation, and detection stages of DON analysis in bulk wheat in a truckload. A dataset of RSD values was obtained across various parameter settings, and the error introduced at each stage was estimated from the average CV value. The overall measurement procedure error was assessed for wheat samples near the DON limit value (1000 ppb), revealing that DON contamination in bulk wheat followed a skewed normal distribution. Considering time efficiency, economic cost, and acceptable error thresholds, the optimal measurement procedure for bulk wheat in transit includes: sampling using a random point method with at least 11 sampling points, with each primary sample weighing 500 g, and three mixing cycles of the composite sample using a cone-shaped divider before subsampling, with a laboratory sample size of 750 g. Samples should be ground to a particle size of 1.0 mm, thoroughly mixed, and 5 g of the homogenized sample should be accurately weighed for analysis. This procedure introduces a relatively low error compared with existing mycotoxin or grain DON detection procedures. This lower error may be partially attributed to the smaller wheat grains, which increase the number of individual kernels per unit mass and improves representativeness relative to larger grains such as corn or peanuts. The largest source of error in the wheat DON measurement procedure is the sampling stage, which aligns with the error source analyses in the aflatoxin measurement procedures for corn and peanuts. The results also revealed that the procedure specified in CXS 193 introduced a higher relative error (20.3%) than the procedure recommended in this study, primarily due to differences in primary sample size. Conversely, the EU 401 recommendation of collecting 100 sampling points would substantially increase time and cost without providing meaningful improvements, as error reduction plateaued once sampling exceeded 11 points. Overall, in this study, we established a DON measurement procedure for wheat in transit that minimizes error, is cost- and time-efficient, and has strong practical applicability. Given that the bulk wheat samples in this study were suspected to contain DON near the regulatory limit, investigations into the concentration gradients were limited. Future research should expand the concentration gradient range to establish a data foundation for constructing OC curves using this optimized procedure.

## 4. Materials and Methods

### 4.1. Investigation of Increments and Sample Size

**Figure 7:**
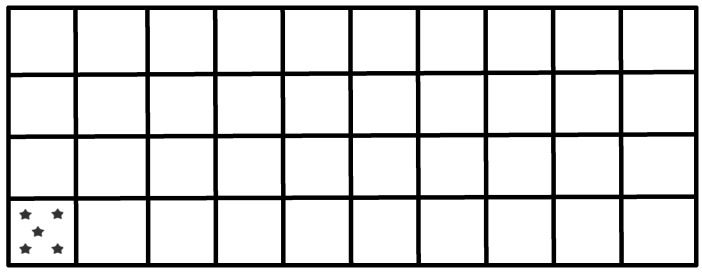
Schematic diagram of sampling in the truck compartment. As shown, the entire compartment is uniformly divided into a 10 × 4 grid. The grid marked with asterisks (★) is presented only as an example; in actual sampling, five sampling points are independently and randomly arranged within each grid. Wheat samples are systematically collected from these points to scientifically determine the appropriate scale of the primary sample size.

A 30-ton bulk wheat truckload, measuring 10 m (length) × 2.5 m (width) × 3.5 m (height), was sourced from Liuxiang Town, Mingguang City, Chuzhou, Anhui Province. A random point sampling method was employed, with 200 sampling points distributed throughout the truckload (Figure 7). At each point, primary wheat samples of 0.1, 0.3, and 0.5 kg were collected. All primary samples were ground using a Retsch mill equipped with a 1.0 mm sieve mesh. The resulting wheat powder was thoroughly mixed and analyzed via liquid chromatography-tandem mass spectrometry (LC-MS/MS).

### 4.2. Study on Mixing Times of Aggregate Samples

**Figure 8:**
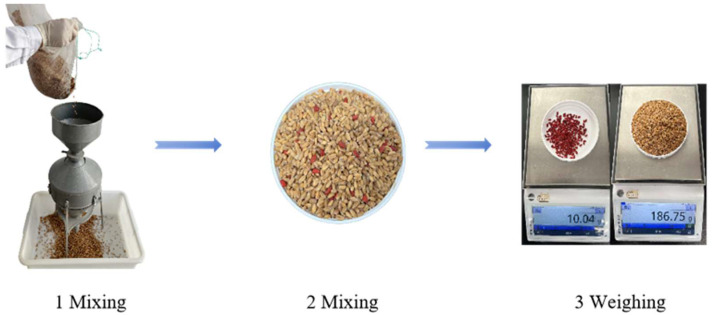
Wheat kernel mixing process. (1) Sample mixing using a cone-shaped divider. (2) Random sampling of the mixed wheat to identify and separate the colored wheat kernels. (3) Weighing the colored and normal wheat to calculate the percentage of colored wheat in the sample.

A 10 kg wheat kernel sample was selected for this study. To evaluate the homogeneity of the wheat mixture, 5% wheat kernels were dyed and strategically placed in the upper, middle, and lower sections of the samples for the mixing studies. The mixture was subjected to one, two, three, and five mixing cycles, with five 500 g wheat samples randomly extracted after each mixing cycle (Figure 8). The proportion of the dyed wheat grains and relative standard deviation were then assessed to determine the uniformity of the mixture.

### 4.3. Investigation of Laboratory Samples

A comparison of domestic and international standard methods for DON detection in wheat was performed to identify differences in laboratory sample size requirements. The Codex Alimentarius CXS 193, ISO 24333, and GB 5009.111 standards specify a laboratory sample size of 1 kg. In contrast, the EU 401 standard [24] requires a sample size of 8–12 kg, depending on the lot size. To determine the minimum representative laboratory sample size, 45 kg of newly harvested wheat was collected from a truckload. The sample was mixed three times according to the method outlined in Section 2.2, and a 7.5 kg portion was randomly extracted and subdivided into 50 units, each weighing 0.05 kg, while the remaining 37.5 kg was divided into 150 units (0.25 kg each). All units were ground using a Retsch 3000 mill (Retsch, Haan, Germany) equipped with a 1.0 mm sieve mesh. The ground samples were manually mixed for 10 min, and 20 g of the powder was weighed for analysis. Measurement data points (1, 2, 3, 4, 5, and 6) were selected randomly using the replacement sampling method. This data acquisition process was repeated 30 times to obtain six datasets. Laboratory sample size datasets of 50, 100, 250, 500, 750, 1000, 1250, and 1500 g were constructed and evaluated to assess the representativeness of the laboratory sample sizes for DON detection in wheat.

### 4.4. Investigation of Grinding Particle Size

A 9 kg wheat sample was collected from wheat grains suspected of DON contamination and was mixed three times according to the method described in Section 2.2. The mixed sample was then evenly divided into three units, each ground using a Retsch 3000 mill equipped with sieve mesh sizes of 1.0, 0.5, and 0.25 mm, respectively. Each ground sample was mixed for 10 min using a V-type mixer and then evenly divided into 200 portions, from which 5 g was accurately weighed for analysis.

### 4.5. Investigation of Test Portions

The minimum weighing volume was evaluated using the 1.0 mm particle size samples obtained in Section 2.4. Each sample was accurately weighed at 1 g, totaling 200 samples. These samples were analyzed using LC-MS/MS(Framingham, Massachusetts, USA). Based on the analytical results of the 1 g samples, datasets representing test portion sizes of 2 g, 5 g, 10 g, and 25 g were generated using a random sampling and combination approach with replacement. This was achieved by merging and averaging 2, 5, 10, and 25 individual 1 g samples, respectively. These datasets were then used to assess the representativeness of different test portion sizes for DON analysis in wheat.

### 4.6. LC-MS/MS Detection Method

LC-MS/MS analysis was performed according to previously reported methods with some modifications [39,40,41]. After weighing the samples in tubes, a certain volume of acetonitrile–water–acetic acid mixture (70 + 29 + 1, *v*/*v*/*v*) was added, followed by shaking on a rotary shaker for 30 min and sequential centrifugation (3500× *g*, 5 min). The supernatant (0.5 mL) was aspirated into a 1.5 mL centrifuge tube, diluted with 0.5 mL water, and shaken on a vortex shaker for 1 min, followed by centrifugation (7200× *g*, 10 min, 4 °C). The supernatant was filtered through a 0.22 µm PTFE filter and an aliquot (180 µL) of the filtrate was combined with 20 µL of stable isotope dilution (SID) working solution prior to injection.

DON was detected and quantified using an HPLC-MS/MS system equipped with a 30A HPLC and Triple Quard 6500 MS/MS system (AB Sciex, Foster City, CA, USA). Separation was performed on a C18 column (2.1 mm × 100 mm, 1.7 μm bead diameter, Waters, Milford, MA, USA) at a column temperature of 40 °C, using a binary mobile phase consisting of MeOH (mobile phase A), 1% acetic acid in water, and 5 mM ammonium acetate (mobile phase B). Gradient elution was performed at a flow rate of 0.3 mL/min as follows: 0 min, 10% A; 2 min, 10% A; 3 min, 20% A; 7 min, 24% A; 10.5 min, 30% A; 13.5 min, 60% A; 15 min, 70% A; 18 min, 75% A; 18.1 min, 95% A; 21.9 min, 95% A; 22 min, 10% A; 25 min, 10% A. The sample injection volume was 2 µL.

MS/MS was performed in positive electrospray ionization (ESI+) mode with multiple reaction monitoring (MRM). The MS source parameters were: ion spray voltage (IS), 4500 V; source temperature (TEM), 600 °C; gas1 (GS1), 55 psi; gas2 (GS2), 50 psi; curtain gas (CUR), 35 psi; collision-activated dissociation gas (CAD), 6 psi. The retention times, ESI modes, and collision energies of the compounds are listed in Table S4.

### 4.7. Statistics and Analysis

Based on the obtained raw detection data, new datasets under different parameter conditions were generated through random sampling with replacement. For each newly generated dataset, two samples were randomly selected to calculate their mean and coefficient of variation (CV). This random sampling and calculation process was repeated 20 times, ultimately constructing a dataset consisting of 20 CV values. Statistical parameters such as the median and mean were then derived from this CV dataset. The entire data processing workflow was implemented using a compact computational program developed in Python (version 3) for this study.

## Figures and Tables

**Table 1:** Errors introduced from the sampling and sample preparation steps.

Methods	Number of Sampling Points and Primary Sample Size	Number of Mixing Cycles/(RSD%)	Laboratory Samples (kg)/(RSD%)	Crushed Particle Size—Weighing Amount (mm-g)/(RSD%)	Relative Error
CXS 193	10 × 100 g (19.9%)	-	1/(2.59)	0.85 mm–25 g (3.0)	20.3
This study	11 × 500 g (8.6%)	3/(6.9)	0.75/(2.77)	1 mm–5 g (5.57)	12.7

## Data Availability

The original contributions presented in this study are included in the article/Supplementary Materials. Further inquiries can be directed to the corresponding authors.

## Supplementary Materials

The following supporting information can be downloaded at: https://www.mdpi.com/article/10.3390/toxins18010013/s1, Table S1. Descriptive Statistical Analysis of the Number of Sampling Points and Primary Sample Size Data; Table S2. Effect of Mixing Times on the Uniformity of Mixed Samples; Table S3. Average and median values corresponding to the box plot of CV values under different crushing particle sizes (%); Table S4. Compound-Dependent Parameters for MRM Mode in LC−MS/MS.
