# Supplementary material for: Systematic Approach to Reducing Errors in Deoxynivalenol Quantification: Insights from Bulk Wheat Sampling and Sample Preparation
Source: Toxins (Basel). 2025 Dec 24;18(1):13. doi: 10.3390/toxins18010013 (PMC12845785; doi:10.3390/toxins18010013)
Supplement: Supplementary file 1 [file toxins-18-00013-s001.zip › toxins-4020979-supplementary.pdf]

Systematic Approach to Reducing Errors in Deoxynivalenol Quantification: Insights from Bulk Wheat Sampling and Sample Preparation

Li Li [1], Bingjie Li [1], Jin Ye [1], Di Cai [1], Yu Wu [1], Peng Li [1], Bing Zhang [1], Jie Wang [2], Xiujuan Li [3], Yi Shao [4,5,*] and Songxue Wang [1,*]

[1] Academy of National Food and Strategic Reserves Administration, NFSRA Key Laboratory of Grain and Oil Quality and Safety, Beijing 100037, China; ll@ags.ac.cn (L.L.); haiyangzhixin2006@163.com (B.L.); yj@ags.ac.cn (J.Y.); cd@ags.ac.cn (D.C.); wyu@ags.ac.cn (Y.W.); lp@ags.ac.cn (P.L.); zb@ags.ac.cn (B.Z.)
[2] College of Food Science and Technology, Nanjing Agricultural University, 1 Weigang Road, Nanjing 210095, China; 2023808109@stu.njau.edu.cn
[3] Grain and Oil Product Quality Supervision and Inspection Station of Xinjiang Uygur Autonomous Region, Urumqi 830049, China; lixj@ags.ac.cn
[4] NHC Key Laboratory of Food Safety Risk Assessment, China National Center for Food Safety Risk Assessment, Beijing 100021, China
[5] School of Public Health, Southern Medical University, Guangzhou 510515, China
* Correspondence: shaoyi@cfsa.net.cn (Y.S.); wsx@ags.ac.cn (S.W.)

Table S1. Descriptive Statistical Analysis of the Number of Sampling Points and Primary Sample Size Data.

Project	100 g	300 g	500 g
Sample size (piece)	200	200	200
Mean (μg/kg)	675.2	740.1	753.8
Standard Deviation (μg/kg)	447.6	314.3	256.2
Min (μg/kg)	46.9	246.6	228.8
25th Percentile (μg/kg)	328.0	507.0	564.7
Median (μg/kg)	538.1	707.6	713.1
75th Percentile (μg/kg)	932.8	902.5	866.3
Max (μg/kg)	2264	2204	1606
RSD (%)	66.3	42.5	34.0

Table S2. Effect of Mixing Times on the Uniformity of Mixed Samples.

Position	Number	AVE（%）	SD（%）	RSD（%）
Bottoms	1	5.06	2.14	42.3
	2	4.32	0.17	3.9
	3	4.44	0.26	5.9
	5	4.59	0.14	3.2
Central Section	1	5.14	0.97	18.9
	2	5.00	0.44	8.8
	3	5.09	0.35	6.9
	5	5.03	0.32	6.4
Upper Section	1	3.57	3.74	104.9
	2	4.99	0.51	10.2
	3	4.88	0.28	5.8
	5	4.81	0.23	4.9

Table S3. Average and median values corresponding to the box plot of CV values under different crushing particle sizes (%).

Particle size	Mean	Median	Q1-Q3（50%）	IQR_bounds(95%)
1mm-5g	5.22	4.29	2.31 - 6.70	0.37 - 13.30
0.5mm-5g	2.98	2.57	0.89 - 3.95	0.39 - 8.54
0.25mm-5g	2.01	1.61	0.98 - 3.01	0.01 - 6.06

Table S4. Compound-Dependent Parameters for MRM Mode in LC−MS/MS.

Compound Name	Retention Time (min)	Precursor ion (m/z)	Product ion (m/z)	Collision Energy (V)
DON	3.12	297.2	249.1Q/203.1C	16/21
^{13}C-DON	3.12	312.2	363.1	15
